# Quantification of Total and Viable Cells and Determination of Serogroups and Antibiotic Resistance Patterns of *Listeria monocytogenes* in Chicken Meat from the North-Western Iberian Peninsula

**DOI:** 10.3390/antibiotics11121828

**Published:** 2022-12-16

**Authors:** Cristina Rodríguez-Melcón, Alexandra Esteves, Sarah Panera-Martínez, Rosa Capita, Carlos Alonso-Calleja

**Affiliations:** 1 Department of Food Hygiene and Technology, Veterinary Faculty, University of León, E-24071 León, Spain; 2 Institute of Food Science and Technology, University of León, E-24071 León, Spain; 3 Department of Veterinary Sciences, School of Agrarian and Veterinary Sciences, University of Trás-os-Montes e Alto Douro (UTAD), 5000-801 Vila Real, Portugal; 4 Veterinary and Animal Research Centre (CECAV), University of Trás-os-Montes e Alto Douro (UTAD), 5000-801 Vila Real, Portugal

**Keywords:** *Listeria monocytogenes*, minced chicken, q-PCR, viability, serogroups, antimicrobial resistance, food microbiology, food safety

## Abstract

Twenty samples of minced chicken meat procured from butcher’s shops in León (Spain; 10 samples) and Vila Real (Portugal; 10 samples) were analyzed. Microbial concentrations (log_10_ cfu/g) of 7.53 ± 1.02 (viable aerobic microbiota), 7.13 ± 1.07 (psychrotrophic microorganisms), and 4.23 ± 0.88 (enterobacteria) were found. The detection method described in the UNE-EN ISO 11290-1 standard (based on isolation from the chromogenic medium OCLA) with confirmation by the polymerase chain reaction (PCR; *lmo1030*) (OCLA–PCR), revealed *Listeria monocytogenes* in 14 samples (70.0% of the total), nine of Spanish origin and five of Portuguese (*p* > 0.05). The levels of viable and inactivated *L. monocytogenes* in the samples were determined with a q-PCR using propidium monoazide (PMAxx) as a viability marker. Seven samples tested positive both with the OCLA–PCR and with the q-PCR, with estimated concentrations of viable cells varying between 2.15 log_10_ cfu/g (detection limit) and 2.94 log_10_ cfu/g. Three samples tested negative both with the OCLA–PCR and with the q-PCR. Seven samples were positive with the OCLA–PCR, but negative with the q-PCR, and three samples tested negative with the OCLA–PCR and positive with the q-PCR. The percentage of viable cells relative to the total ranged between 2.4% and 86.0%. Seventy isolates of *L. monocytogenes* (five from each positive sample) were classified in PCR serogroups with a multiplex PCR assay. *L. monocytogenes* isolates belonged to serogroups IIa (52 isolates; 74.3%), IIc (7; 10.0%), IVa (2; 2.9%), and IVb (9; 12.9%). The susceptibility of the 70 isolates to 15 antibiotics of clinical interest was tested. The strains presented resistance to between three and eight antibiotics. The average number of resistances was greater (*p* < 0.001) among strains isolated from Spanish samples (6.20 ± 1.08), than in those from Portugal (5.00 ± 1.08). In both groups of strains, a prevalence of resistance higher than 95% was observed for oxacillin, cefoxitin, cefotaxime, and cefepime. The need to handle minced chicken meat correctly, taking care to cook it sufficiently and to avoid cross-contamination, so as to reduce the danger of listeriosis, is emphasized. A combination of culture-dependent and culture-independent methods offers complementary routes for the detection in food of the cells of *L. monocytogenes* in various different physiological states.

## 1. Introduction

World per capita consumption of poultry stood at 15.2 kg in 2017, only exceeded by pork (15.7 kg) [1]. This high level of consumption of poultry products may be attributed to its variety, versatility, and low-fat content. Moreover, it is an inexpensive food, easily cooked, offers pleasant sensory qualities, and is acceptable to almost all cultures and religions [2].

A certain proportion of meat is eaten in the form of meat preparations. Regulation (EC) 853/2004 defines meat preparations (e.g., minced meat), as fresh meat, including meat that has been reduced to fragments, which has had foodstuffs, seasonings or additives added to it, or which has undergone processes insufficient to modify the internal muscle fibre structure of the meat, and thus to eliminate the characteristics of fresh meat [3]. Meat preparations are suitable for a range of cooking techniques, and thus satisfy the demands of consumers, who prefer meat products ready to cook, since saving time in the preparation of food has become a priority for most families [4,5].

The considerable consumption of poultry is a good cause for taking an interest in ensuring that any products of this nature offered for sale are safe and have an appropriate texture, flavour, color, and general appearance. Items excessively contaminated with microorganisms are undesirable both financially and from the perspective of Public Health [6].

The muscle of a healthy, living animal is essentially sterile, but even under conditions of strict hygiene it can become contaminated with pathogenic or spoilage bacteria during slaughter and processing [7]. There are several groups of microorganisms, such as viable aerobic microbiota, psychrotrophic bacteria, and enterobacteria, whose counts in meat allow an evaluation of its microbiological safety, the hygiene conditions during processing, any spoilage of products, and their remaining shelf-life [6,8].

*Listeria monocytogenes* is a Gram-positive bacterium in the shape of a bacillus, a facultative anaerobe, and is psychrotrophic and not spore-forming. This microorganism is responsible for listeriosis, an infection whose main route of transmission to humans is via contaminated foodstuffs [9,10]. Each year there are some 23,000 cases of invasive listeriosis worldwide [11]. In the European Union, 1876 cases of invasive listeriosis were recorded in 2020, with a notification rate of 0.42 cases per 100,000 inhabitants and a mortality rate of 13.0%, the highest among all food-borne illnesses [12]. These facts make listeriosis one of the most serious bacteria which is transmitted in foodstuffs. Although 13 serotypes of *L. monocytogenes* have been described, only three (1/2a, 1/2b, and 4b) have been associated with more than 98% of human listeriosis cases [13,14]. Strains from group 1/2 have been associated with sporadic cases of listeriosis, while the serotype 4b is responsible for most outbreaks of disease [15,16]. Therefore, serotype designation is associated with virulence potential.

Official standard methods for detecting pathogenic microorganisms, like *L. monocytogenes* in foodstuffs, are based on enrichment and culturing in a selective medium. They have some drawbacks, such as their long analysis times and problems with the presence of bacterial cells that are viable but not culturable, because these cells would not be detected with such culturing methods [17]. Several speedier techniques, like the quantitative polymerase chain reaction (q-PCR), are effective alternatives for the detection and quantification of pathogenic microorganisms in foods. Furthermore, if they are used with a viability marker, it becomes possible to quantify exclusively viable cells, not just the overall total of cells.

A phenomenon observed over recent years is the increase occurring in the resistance of bacteria to antibiotics, involving not only all the principal pathogenic microorganisms, but also a wide range of antimicrobial substances. The presence in food of bacteria resistant to antibiotics is a worrying matter, in view of the chance of an infection occurring either through handling contaminated foodstuffs, or through eating them when they are inadequately cooked or when there has been cross-contamination. Furthermore, bacteria resistant to antimicrobials may constitute a reservoir of resistance genes transferrable to other bacteria in the food chain [18]. 

It is estimated that within three decades infections by bacteria resistant to antibiotics will become the principal source of mortality, causing some ten million deaths per year worldwide [19,20]. To grasp the magnitude of this problem, these figures must be compared with the 700,000 deaths attributable to antibiotic resistance that occurred in 2014 [19,20]. The financial consequences of resistance to antibiotics are also very heavy; these infections having been estimated to cost the health systems of the United States and the countries forming the European Union of some EUR 1.1 thousand million every year [21]. 

The objective of this research work was to determine the prevalence, levels of total and viable cells, serogroups, and patterns of resistance to antibiotics of *L. monocytogenes* from samples of minced chicken from the north-western of the Iberian Peninsula. This study also determined the amounts of several groups of microorganisms that are indicators of hygiene standards. In order to reveal any differences between countries, the samples were obtained from both Spain and Portugal.

## 2. Results

### 2.1. Levels of Microorganisms Indicating Quality of Hygiene

The average levels (log_10_ cfu/g) found were 7.53 ± 1.02 for viable aerobic microbiota, 7.13 ± 1.07 for psychrotrophic microorganisms, and 4.23 ± 0.88 for enterobacteria (Table 1). No statistically significant difference (*p* > 0.05) was observed between the two countries with respect to viable aerobic microbiota, with 7.81 ± 0.85 log_10_ cfu/g found in Spain and 7.29 ± 1.12 log_10_ cfu/g in Portugal. The same was true for enterobacteria, the Spanish value being 4.55 ± 0.96 log_10_ cfu/g and the Portuguese 4.02 ± 0.78 log_10_ cfu/g. However, samples acquired in Portugal presented lower (*p* < 0.05) levels of psychrotrophic microorganisms, at 6.64 ± 1.10 log_10_ cfu/g, than those procured from Spanish sources, at 7.56 ± 0.86 log_10_ cfu/g. 

Counts for viable aerobic microbiota were similar (*p* > 0.05) to those for psychrotrophic microorganisms. This was true both if the samples from each country were taken separately and if all the samples studied were treated as a single set. Moreover, levels of enterobacteria were lower (*p* < 0.05) than those of the other groups of microorganisms in all cases.

Psychrotrophic microorganisms represented 56.2% of total viable aerobic counts in the samples procured in Spain, 22.4% in those acquired in Portugal, and 39.8% in the set of samples analyzed as a whole.

### 2.2. Prevalence and Levels of Listeria monocytogenes

Colonies with a typical *L. monocytogenes* morphology, greenish blue with a halo on the OCLA medium, were isolated from 14 samples, nine from Spain and five from Portugal. All the colonies isolated, a total of 70, comprising five from each positive sample, were identified as *L. monocytogenes* by PCR. Thus, the prevalence of viable culturable cells of *L. monocytogenes* was 70.0% overall, 90.0% in the samples acquired in Spain and 50.0% in those from Portugal (*p* = 0.070).

**Figure 1:**
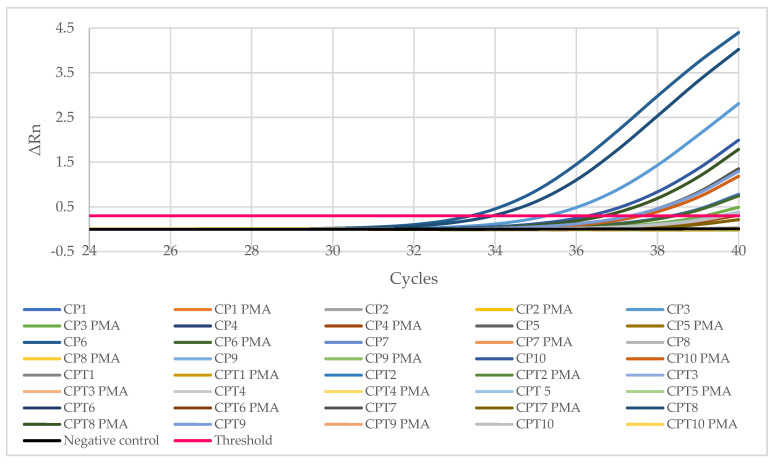
Chart of the amplification of samples of *Listeria monocytogenes* with q-PCR. All twenty samples of minced chicken, with and without the PMAxx marker, are included, together with a negative control, and the value for the threshold of fluorescence.

The samples of minced chicken meat were examined by q-PCR. In each amplification cycle of the q-PCR the quantity of DNA in the sample is doubled. The larger the amount of bacterial DNA present in a sample, the fewer the number of amplification cycles will be necessary for detecting it. A sample is deemed positive when it goes above the fluorescence threshold, set at a value of 0.3. At this point, the equipment indicates a value for Ct, the cycle in which this value is exceeded (Figure 1).

Seven samples, four from Spain and three from Portugal, tested positive with both the OCLA–PCR (isolation from chromogenic medium OCLA and confirmation by PCR) and the q-PCR. Three samples from Portugal yielded negative results with both the OCLA–PCR and q-PCR. Seven samples, five from Spain and two from Portugal, showed positive with the OCLA–PCR, but negative with the q-PCR. Three samples, one from Spain and two from Portugal, tested negative with the OCLA–PCR, but positive with the q-PCR. Thus, the number of samples with *L. monocytogenes* was 14 (considering the OCLA–PCR), 10 (q-PCR), or 17 (combining the results of both culture-dependent and culture-independent methods).

Table 2 shows the results obtained from amplification in the q-PCR in terms of Ct, ng of DNA in the reaction tube and log_10_ cfu/g in the sample, recording both total cells and viable cells. The levels of contamination by *L. monocytogenes* ranged between <2.15 log_10_ cfu/g (limit of detection) and 4.32 log_10_ cfu/g. For the viable cells, concentrations varied between <2.15 log_10_ cfu/g and 3.25 log_10_ cfu/g. The percentage of viable *L. monocytogenes* cells relative to the total lay between 2.4% and 86.0%. 

The conventional OCLA–PCR method and the q-PCR technique were compared with respect to their capacity to detect *L. monocytogenes*. The classic method was taken as the reference technique. Use of the q-PCR yielded values for sensitivity (the ability to pick up positive instances), specificity (the capacity to detect negative cases), and efficiency (the probability of results being correct) of 50.0%. The predictive value for a positive test was 70.0%, and for a negative it was 30.0%. Finally, agreement, in terms of the kappa coefficient, was 0.0.

### 2.3. Serogroups of Listeria monocytogenes

A total of 70 isolates of *L. monocytogenes*, five from each of the samples yielding positive on the OCLA medium and confirmed by PCR, were classified in PCR serogroups with a multiplex PCR assay. The strains were distributed across four serogroups: IIa (52 isolates; 74.3% of total), IIc (7; 10.0%), IVa (2; 2.9%), and IVb (9; 12.9%). In Spain, figures were 84.4% (serogroup IIa), 4.4% (IIc), and 11.1% (IVb). Data from Portugal were 56.0% (IIa), 20.0% (IIc), 8.0% (IVa), and 16.0% (IVb).

### 2.4. Susceptibility to Antibiotics of Listeria monocytogenes

**Figure 2:**
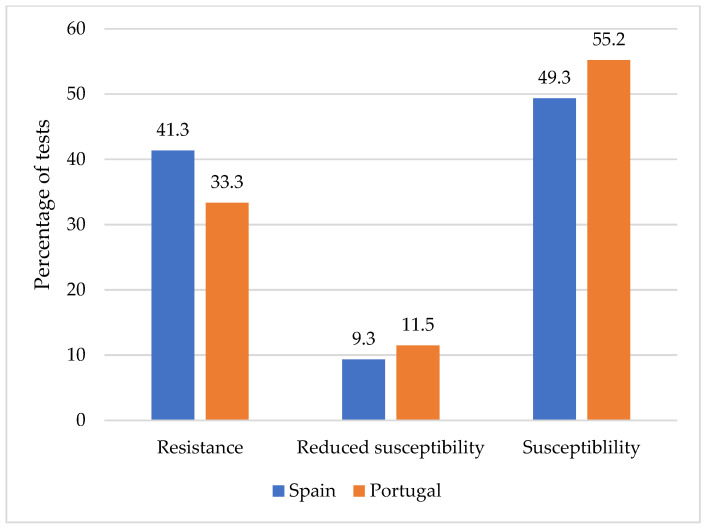
Percentages of tests recording resistance, reduced susceptibility, and susceptibility in strains of *Listeria monocytogenes* from Spanish and Portuguese minced chicken samples.

The 70 *L. monocytogenes* isolates were analyzed to determine their susceptibility to a panel of 15 antibiotics of veterinary and human clinical importance. In all, 1050 tests were undertaken, which was the product of the numbers of the sets of strains and the antibiotics tested. No differences (*p* > 0.05) were observed between the two countries in the percentage of tests in which resistance, reduced susceptibility, and susceptibility were recorded (Figure 2).

All the strains were multi-resistant to between three and eight antibiotics. The average number of resistances per isolate was 5.77 ± 1.22 overall. This average was higher (*p* < 0.001) among strains isolated in Spain, at 6.20 ± 1.08, than in those from Portugal, for which the figure was 5.00 ± 1.08. If resistance and reduced susceptibility are taken together, the figures are 7.29 ± 1.16 for the whole set of strains, 7.60 ± 1.10 for strains of Spanish origin, and 6.72 ± 1.06 for strains from Portugal. 

Table 3 displays the patterns of resistance detected in the 70 strains of *L. monocytogenes* studied. The most commonly observed pattern was resistance to OX-FOX-CTX-FEP-CIP-F, present in 14 strains (10 Spanish and 4 from Portugal). Other phenotypes seen with some frequencies were OX-FOX-CTX-FEP-CIP, noted in seven strains of Spanish origin and six from Portugal, and OX-FOX-CTX-FEP, seen in seven strains from Spain and one from Portugal. 

**Figure 3:**
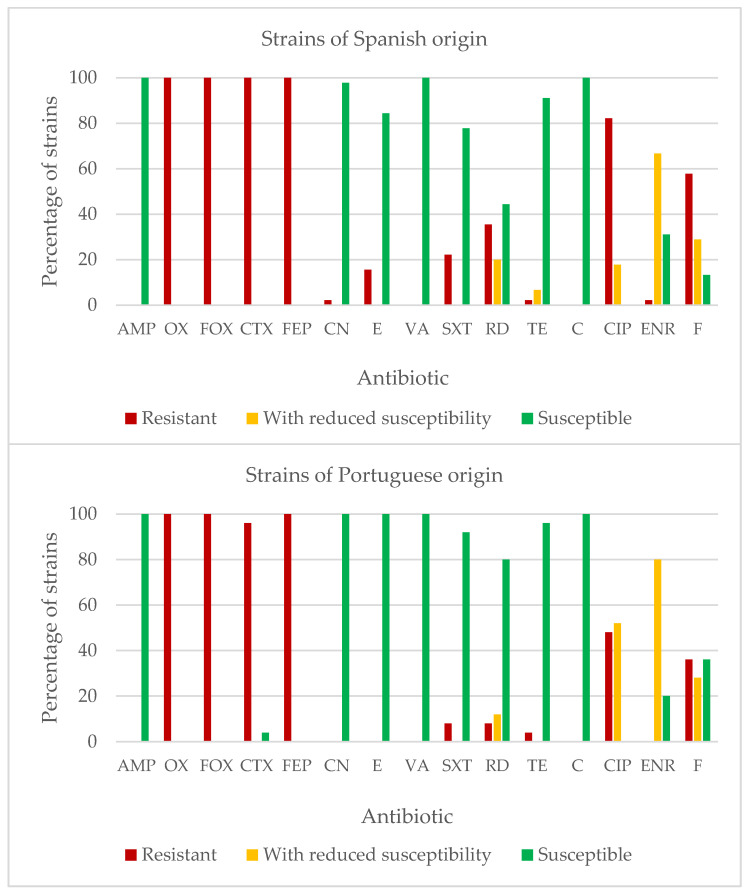
Percentages of strains of *Listeria monocytogenes* which are either resistant, have reduced susceptibility, or are susceptible to each antibiotic tested: AMP (ampicillin, 10 µg), OX (oxacillin, 1 µg), FOX (cefoxitin, 30 µg), CTX (cefotaxime, 30 µg), FEP (cefepime, 30 µg), CN (gentamycin, 10 µg), E (erythromycin, 15 µg), VA (vancomycin, 30 µg), SXT (trimethoprim-sulfamethoxazole, 25 µg), RD (rifampicin, 5 µg), TE (tetracycline, 30 µg), C (chloramphenicol, 30 µg), CIP (ciprofloxacin, 5 µg), ENR (enrofloxacin, 5 µg), and F (nitrofurantoin, 300 µg).

Figure 3 shows the percentages of strains of *L. monocytogenes* resistant to each of the antibiotics considered. The results were similar for the two countries, with percentages of resistant strains in excess of 95.0% in oxacillin, cefoxitin, cefotaxime, and cefepime. On the other hand, percentages of resistant strains below 25.0% were observed for the following antibiotics (percentages in brackets are for Spain and Portugal, respectively): ampicillin (0.0% and 0.0%); gentamycin (2.2% and 0.0%); erythromycin (15.6% and 0.0%); vancomycin (0.0% and 0.0%); trimethoprim-sulfamethoxazole (22.2% and 8.0%); tetracycline (2.2% and 4.0%); and chloramphenicol (0.0% and 0.0%). For the remaining antibiotics, intermediate percentages of resistance were noted in Spain and Portugal, respectively: ciprofloxacin (17.8% and 52.0% strains), rifampicin (20.0% and 12.0%), and nitrofurantoin (28.9% and 28.0%).

When the strains with resistance and with reduced susceptibility were grouped together as a single cohort, a value of 100% of resistant strains, in both Spain and Portugal, was recorded for oxacillin, cefoxitin, and cefepime. Other percentages found for this combined grouping for Spain and Portugal, respectively, were 100% and 96.0% for cefotaxime; 55.6% and 20.0% for rifampicin; 22.2% and 8.0% for trimethoprim-sulfamethoxazole; 2.2% and 0.0% for gentamycin; 15.6% and 0.0% for erythromycin; and, finally, 8.9% in Spain and 4.0% in Portugal for tetracycline. No strains presented any resistance or reduced susceptibility to ampicillin, vancomycin, or chloramphenicol.

The strains isolated in Spain presented a greater prevalence of resistance to erythromycin (*p* < 0.001), trimethoprim-sulfamethoxazole (*p* < 0.01), rifampicin (*p* < 0.001), ciprofloxacin (*p* < 0.001), and nitrofurantoin (*p* < 0.001). Only for gentamycin was a greater prevalence of resistance noted in the strains isolated in Portugal (*p* < 0.001). The place of origin of the strains had no influence on the prevalence of resistance to the remaining antibiotics tested.

When the strains with resistance were grouped together with those having reduced susceptibility, the prevalence was higher in Spain with regard to rifampicin (*p* < 0.001), trimethoprim-sulfamethoxazole (*p* < 0.01), and erythromycin (*p* < 0.001). In contrast, the strains isolated in Portugal presented a greater percentage of resistance or reduced susceptibility to ciprofloxacin (*p* < 0.001) than those from Spain.

**Figure 4:**
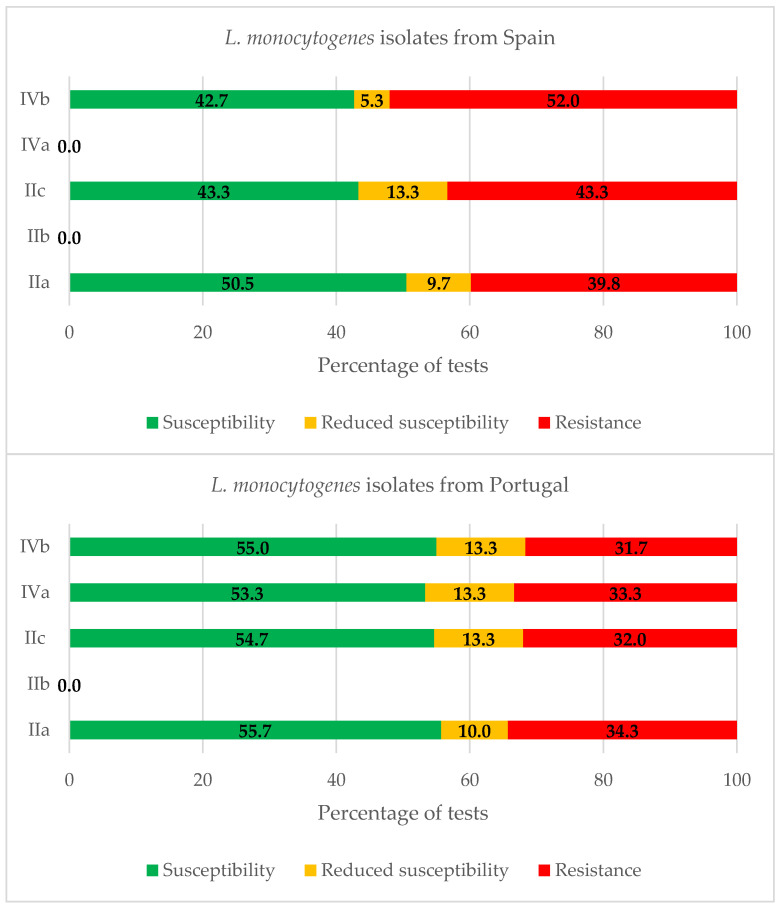
Percentages of strains of *Listeria monocytogenes* which are either resistant, have reduced susceptibility, or are susceptible in each serogroup.

Figure 4 shows the percentages of reactions which are either resistant, intermediate, or susceptible for the strains from Spain and Portugal in each serogroup. Considering simultaneously the isolates with resistance or reduced susceptibility, the average values of 48.1%, 48.6%, 46.7%, and 51.9% were observed for serogroups IIa, IIc, IVa, and IVb, respectively.

## 3. Discussion

### 3.1. Levels of Microorganisms Indicating the Quality of Hygiene

The study being reported here investigated the microbiological quality of samples of minced chicken procured from various retail outlets in Spain and Portugal. For this purpose, the levels, expressed in log_10_ cfu/g, of viable aerobic microbiota, of psychrotrophic microorganisms, and of enterobacteria were determined.

Counts of viable aerobic microbiota are widely used to estimate overall microbial contamination of foodstuffs. Although high levels of these microorganisms do not necessarily imply a potential risk for human health, their importance lies in the fact that they are indicators of the quality of hygiene in the areas where food is processed and of the products themselves [6]. The viable aerobic microbiota count has been utilized as a parameter in predicting the shelf life of meat, since the presence of these microbes in large quantities can trigger rapid spoilage of products [22]. These microorganisms can also act as indicators of inappropriate processing, so that quantifying them is one way of monitoring good manufacturing practices (GMPs). 

Guidelines and recommendations have been drawn up to check the microbial quality of meat preparations. According to the norms for GMPs, the overall level of microbiological contamination (viable aerobic microbiota) in raw meat preparations should not exceed 5, or at the very most 7 log_10_ cfu/g [23,24], figures which are lower than those noted in the present research. Moreover, a formula for sampling three categories for viable aerobic microbiota in mince at the end of the manufacturing process, n = 5, c = 3, m = 5.70 log_10_ cfu/g, and M = 6.70 log_10_ cfu/g, is in application at the current time in the European Union [25]. In accordance with the criteria stated, “n” is the number of units making up the sample and “c” is the number of units in a sample in which values higher than “m”, but never going above “M”, are permitted. The results are deemed satisfactory if all the values observed (n) are below m, acceptable if a maximum of the c values lie between the m and M, and unsatisfactory if any value exceeds M or if more than the c values go above m. The average figures noted in the present study surpassed the value of M. Hence, on the basis of the microbiological criteria noted, it may be stated that the counts recorded are excessively high.

The levels, expressed in log_10_ cfu/g, of viable aerobic microbiota in the samples, at 7.81 ± 0.85 for those acquired in Spain and 7.29 ± 1.12 for those from Portugal (*p* > 0.05), were higher than those previously recorded in poultry in Spain, which ranged from 6.29 ± 0.64 to 7.28 ± 0.51 [4,8]. They were also higher than in other zones in the European Union, such as France, where a figure of 6.05 ± 0.18 was noted [26]. Such high levels, especially in the samples procured in Spain, may be due to inappropriate processing or a lack of cleanliness in the equipment and installations utilized. With reference to this, it should also be emphasized that the mincing of meat contributes to its contamination, as a consequence of touching different surfaces, modification to the structure of tissues, the increase in the surface area, and the contact with air as chopping occurs [27]. Furthermore, in the present study, the time elapsed since slaughter, at the very least several days, may to some extent be an explanation for the high counts recorded [28]. 

Psychrotrophic microorganisms are of particular relevance when products are kept under refrigeration because such storage conditions still allow them to multiply [22]. The results from the current study fall within the broad range of values, running from 3.5 to 10.7 log_10_ cfu/g, recorded by other authors [29] for poultry products, including mince. Nevertheless, the average counts in log_10_ cfu/g for psychrotrophic microorganisms observed in this work, at 7.13 ± 1.07, are higher than those previously encountered by members of the same research group in samples of poultry preparations, at 6.66 ± 1.09 [4], in chicken carcasses, at 4.84 ± 0.60 [30], and in chicken legs, ranging between 4.34 ± 0.77 and 7.07 ± 1.07 [6,8]. It must be noted that all the samples in this research exceeded the maximum limit established by Pascual-Anderson [31] for poultry in Spain, which stands at 5 log_10_ cfu/g. It is probable that the levels of psychrotrophic microorganisms grew relative to the initial counts during the time spent in refrigerated storage in the shop prior to purchase, as previously noted [4,6]. 

Differences (*p* < 0.05) were observed between the counts of psychrotrophic microorganisms recorded in samples of chicken mince from Spain, at 7.56 ± 0.86, and from Portugal, at 6.64 ± 1.10. The psychrotroph counts in the samples of mince acquired in Portugal were close to the levels previously seen in turkey preparations, with a figure of 6.27 ± 1.17 log_10_ cfu/g being quoted by Castaño-Arriba et al. [32]. It should be noted that none of the samples acquired showed signs of spoilage, even though some researchers [33] have stated that levels of this group of microbes of between 6 and 8 log_10_ cfu/g are enough to change the smell and appearance of meat. 

The low percentage of psychrotrophic microorganisms relative to viable aerobic microbiota, standing at 39.8% for the set of samples as a whole, and especially in the samples procured in Portugal, at just 22.4%, suggests that there were irregularities in maintaining temperatures at some point during the processing, storage, transport, distribution, or display in the shops of these products, as suggested previously [28]. In other studies of refrigerated chicken, the concentration of psychrotrophic microorganisms was higher than that of viable aerobic microbiota [30,34,35].

Most of the enterobacteria found in fresh meat come from contamination with faeces, so that their presence in large quantities may point to poor hygiene at the abattoir from which the product originates, inappropriate storage, or a combination of both [34,36,37]. Hence, the level of enterobacteria has been used as an indicator of faecal contamination in fresh meat. The average counts for enterobacteria in the present study, at 4.23 ± 0.88 log_10_ cfu/g, lay above the limits used as microbiological criteria for free-range poultry in Spain, which are set at 2 log_10_ cfu/g [31]. 

No significant differences were observed for enterobacteria as a group between samples analyzed in Spain, with 4.55 ± 0.96 log_10_ cfu/g, and in Portugal, with 4.02 ± 0.78 log_10_ cfu/g. Various studies refer to lower levels of enterobacteria in poultry, both in Spain, where a figure of 2.89 ± 0.77 log_10_ cfu/g was recorded by Buzón-Durán et al. [4], and in other areas within the European Union, with 2 log_10_ cfu/g noted by Fraqueza et al. [38]. Previous studies also carried out in north-western Spain [3] indicated a lower load of enterobacteria in meat preparations based on beef, at 1.99 ± 0.99 log_10_ cfu/g, and pork, at 1.96 ± 1.44 log_10_ cfu/g. This greater count of enterobacteria in poultry products when compared to foodstuffs of other types may have been an outcome of the higher initial pH in birds’ muscle meat [39].

### 3.2. Prevalence and Levels of Listeria monocytogenes

It proved possible to assign 100% of the colonies with the characteristic morphology of *L. monocytogenes* on OCLA medium to that species using the PCR technique, detecting the *lmo1030* gene. These results coincide with those of several other studies, in which a comparison of the classic method of double enrichment and inoculation onto a solid medium with a PCR approach for detecting *L. monocytogenes* found figures for agreement between the two ranging from 80% to 100% [40,41,42]. 

The strains of *L. monocytogenes* were isolated from 70.0% of the samples of minced chicken. They were found in 90.0% of the samples obtained from Spanish outlets and in 50.0% of those from Portugal. This prevalence falls within the broad range of values recorded by other authors, with between 0% and 99% of samples of poultry testing positive for *Listeria* spp. [43]. In previous work done by members of the research group undertaking this study, the prevalence observed for *L. monocytogenes* was 24.5% [44] or 32% [30], much lower than the values from the present case. Nevertheless, it must be noted that those other research studies investigated either chicken carcasses or chicken legs. As the mincing of meat increases the risk of contamination by *L. monocytogenes* [45], it was to be expected that the prevalence found in the current work would be higher than in previous investigations. With regard to the prevalence of *L. monocytogenes* in samples of food in Portugal, the value noted, 50.0%, is similar to the range observed by other authors in raw chicken in that country, with published prevalence values of 19.3% [46], 41.3% [47], or 60% [48].

In the seven samples that tested positive with both OCLA–PCR and q-PCR, estimated concentrations ranged between 2.25 and 4.32 log_10_ cfu/g. Such levels of contamination are similar to those observed by other authors in raw chicken, where the detected values did not exceed the value of 3 log_10_ cfu/g of sample [49]. Other researchers have found concentrations above 3 log units, but only in a small percentage of samples [50]. The results of the present study are also similar to what was encountered by other authors in fermented sausages, where the range was 2.85 to 3.38 log_10_ cfu/g [51], or in fresh cheese, for which 3.60 log_10_ cfu/g was the figure quoted [52]. 

According to the model utilized by the European Food Safety Authority (EFSA), 92% of cases of invasive listeriosis are to be attributed to doses of more than 5 log_10_ cfu per portion consumed. If the average size of a portion is taken to be 50 g, this would equate to a concentration of *L. monocytogenes* in ready-to-eat foods in excess of 3.30 log_10_ cfu/g at the point of consumption. Nonetheless, a small proportion of cases are associated with such foodstuffs having lower levels than this of *L. monocytogenes* [53]. Regulation (EC) num. 2073/2005 [25] establishes as the upper limit of shelf life a concentration of 2 log_10_ cfu/g in ready-to-eat foods on sale. 

It is true that chicken preparations are cooked before consumption. However, the risk arising from the presence of viable cells of *L. monocytogenes* in such foodstuffs, especially when it is at a high level, derives from the possibility of inadequate cooking or of incidents of cross-contamination affecting other foods. Moreover, *L. monocytogenes* is a psychrotrophic microorganism, so that its concentration in mince can grow over the course of refrigerated storage. 

Three samples of minced chicken tested negative both with the classic OCLA–PCR isolation method and with the q-PCR. Either these samples had no *L. monocytogenes*, or they were contaminated only with cells that were inactivated, or viable, but not culturable (and at a concentration lower than the detection limit for the q-PCR technique, 2.15 log_10_ cfu/g). If culturable cells were present in the samples, their concentration was below 1 ufc/25 g. Seven samples tested positive with the OCLA–PCR and negative with the q-PCR. This may have been due to the fact that 40 amplification cycles, equating to 2.15 log_10_ cfu/g, taken as the limit for detection by the technique, were not enough to get over the fluorescence threshold. Hence, these samples were deemed to be contaminated by viable culturable cells of *L. monocytogenes*, but at a concentration below 2.15 log_10_ cfu/g. It should be noted that the detection limit adopted was lower than those set by other authors, lying between 3 and 4 log_10_ cfu/g or cfu/mL [52,54,55]. Finally, three samples tested negative with the OCLA–PCR and positive with the q-PCR, which may be an outcome of the fact that the cells of *L. monocytogenes* were viable but not culturable or that the DNA came from inactivated bacteria. If culturable cells were present, their concentration was below 1 ufc/25 g. 

The limited sensitivity of the q-PCR with regard to the OCLA–PCR (50.0%) shows clearly that this method is not of any use in detecting *L. monocytogenes* in food when contamination levels are very low, because of the detection threshold for the technique, at 2.15 log_10_ cfu per gram of sample. Hence, the predictive value of a negative test is no more than 30.0%. In contrast, the q-PCR does have the advantage over the classic method of allowing detection of cells inactivated and cells that are viable, but not culturable. This is what leads to the low specificity of the technique (50.0%), since the classic method used as a benchmark rate as negative any samples contaminated with *L. monocytogenes* cells that are not culturable or are inactivated. In particular, three samples in the present study gave evidence of the detection by the q-PCR (using the viability marker PMAxx) of viable *L. monocytogenes* cells that could not be cultured on OCLA medium. The detection and quantification of cells that are viable, but not culturable, is of interest in the context of food safety, since these are living cells that could cause infections in consumers.

The percentages of viability recorded in this research varied between 2.4% and 86.0%. A search of published works did not find any research into *L. monocytogenes* allowing a comparison of these percentages. The results do lie within the range of values obtained by Zhang et al. [56] for other pathogenic Gram-positive microorganisms like *Staphylococcus aureus*, the concentration of viable cells of which in powdered milk was 10% (3 × 10^2^ cfu/g relative to a total 3 × 10^3^ cfu/g).

### 3.3. Serogroups of Listeria monocytogenes

The serogroup IIa was the most prevalent in minced chicken meat from both Spain and Portugal (84.4% and 56.0% of isolates, respectively). The strains in this serogroup (which includes serotype 1/2a) have shown extensive distribution in foodstuffs and food-processing environments around the world, thereby indicating its robust ecological adaptability [57,58,59,60,61,62]. It has been indicated that the strains of the dominant serovar from this serogroup (serotype 1/2a) appears to contain more plasmids than other serotypes. Considering that plasmids frequently carry genes that confer resistance to antimicrobial agents, including sanitizers used in processing operations, bacteria harbouring such plasmids would have an increased ability to survive and develop in these environments [16]. Moreover, isolates from serotype 1/2a present a high prevalence of various virulence genes and are often isolated from human clinical samples [63].

The strains belonging to serogroups IIc (including serotype 1/2c) and IVb (including serotype 4b), which were detected in the study being processed here, have been previously detected in food samples [64,65,66]. The presence of *L. monocytogenes* isolates from serogroup IVb is a matter of concern because the serotype 4b strains exhibit the strongest epidemiological association with human listeriosis [67]. It should be noted that no strains from serogroup IIb were found in minced chicken meat, which does not coincide with results from most authors consulted. It has been suggested that differences in geographical region and in the investigated type of food may cause variations in serogrouping [67]. On the other hand, serogroup IVa (strains 4a–4c), to which 8.0% of isolates from Portugal belonged, is considered very infrequent in foodstuffs, and its presence is linked exclusively to animals. The strains of this serogroup are rarely reported with clinical relevance for humans [59].

### 3.4. Susceptibility of Antibiotics

The susceptibility of 70 isolates of *L. monocytogenes* obtained from chicken mince was checked against 15 antibiotics of clinical significance. The average number of resistances per isolate (5.77 ± 1.22; 6.20 ± 1.08 for the Spanish strains and 5.00 ± 1.08 for those from Portugal), was much higher than the values recorded in previous work with the strains of *L. monocytogenes* obtained from poultry in north-western Spain, in which the number of resistances per strain observed was 1.60 in 1993 and 4.24 in 2006 [44]. Although *L. monocytogenes* is a bacterium that in the past has been sensitive to the majority of antimicrobials employed to treat infections by Gram-positive organisms, in recent years a striking increase has occurred in the prevalence of resistance in this microorganism [68], a situation that is also highlighted by the results of the present study, where the strains showed resistance to antibiotics used for the treatment of human listeriosis (e.g., erythromycin, trimethoprim-sulfamethoxazole, fluoroquinolones, and rifampicin) [16]. Among other causes, this growth in resistance in *L. monocytogenes* is due to its gradual acquisition from the cells of various different genera of bacteria of mobile genetic elements, such as plasmids or transposons [69]. It should be noted that resistance to antibiotics has been commonplace for some years in other Gram-positive bacteria. Thus, the average number of resistances per strain noted in this work is similar to what was found previously for Gram-positive bacteria in poultry from north-western Spain, where figures of 4.50 [32] or 5.58 [70] were observed for enterococci, and 6.35 for *S. aureus* [4].

A group of international experts established under a joint initiative of the European Centre for Disease Prevention and Control (ECDC) and the Centers for Disease Control and Prevention (CDC) in the United States devised the standard definitions for phenotypes, which were seen as multidrug-resistant (MDR), extensively drug-resistant (XDR), and pan-drug-resistant (PDR) in bacteria of interest for Public Health. The MDR phenotype is defined as acquired non-susceptibility to at least one agent in three or more antimicrobial categories, with one or more antibiotics from each category being applied. The XDR phenotype is defined as non-susceptibility to at least one agent in all but two or fewer antimicrobial categories, so that the bacterial isolates remain susceptible to only one or two categories. Finally, the PDR phenotype refers to a lack of susceptibility affecting all agents in all antimicrobial categories [71]. These criteria were used in characterizing the profile of resistance to antibiotics on the part of the strains trialled in the present study.

No pan-susceptible strains were found, nor any that were resistant to just one antibiotic. One strain (1.4% of the total) showed resistance to three antibiotics, eight strains (11.4%) to four antibiotics, twenty-three strains (32.9%) to five, and thirty-eight strains (54.3%) presented a multidrug-resistant phenotype (MDR). The isolates assigned to the MDR category presented resistance to six (20 strains; 28.6% of the total), seven (10 strains; 14.3%), or eight (8 strains; 11.4%) antimicrobial compounds. The presence of strains with resistance to various antibiotics constitutes a crucial challenge for Public Heath because many antimicrobials would consequently be ruled out as therapeutic options [18].

More than 50% of the strains presented resistance to oxacillin, cefoxitin, cefotaxime, cefepime, ciprofloxacin, enrofloxacin, and nitrofurantoin. The strains of Spanish origin showed, in addition, intermediate levels of resistance to tetracycline (6.7% of strains), rifampicin (20.0%), ciprofloxacin (17.8%), enrofloxacin (66.7%), and nitrofurantoin (28.9%). These findings are worrying, since some of the substances mentioned are habitually employed in treating listeriosis, for which a beta-lactam, generally ampicillin, would be the antibiotic of choice, administered alone or in combination with gentamycin. Where an allergy to beta-lactams occurs, possible alternatives include erythromycin, vancomycin, trimethoprim-sulfamethoxazole, and fluoroquinolones. Occasionally, treatment for listeriosis is undertaken with rifampicin, tetracycline, and chloramphenicol [16].

Furthermore, certain substances to which the strains in the present study showed a high prevalence of resistance are classified by the World Health Organization as antimicrobial agents that are “critically important” (AMP, CIP, ENR, FOX, CTX, FEP, and RD), “highly important” (OX and SXT), or “important” (F) for human medicine [72]. It should also be noted that the other antibiotics to which the strains studied presented some resistance, even if to an extent below 15%, are classified as “critically important” (CN, E, and VA) or “highly important” (TE) in human medicine. In the list published by the World Organization for Animal Health (OIE), AMP, OX, CIP, ENR, SXT, CN, and TE are considered antibiotics that are “critically important”, and RD is “highly important” in veterinary medicine [73]. 

Various authors have found the strains of *L. monocytogenes* resistant to one or more of the antibiotics to which resistance was noted in the current study [16,44,69,74,75,76,77,78]. It must be noted that some of the antibiotics to which resistance was observed are substances that are widely used in animal production (e.g., fluoroquinolones) [79,80,81]. Hence, the selective pressure exerted by the use of antibiotics (particularly when incorrectly employed at sub-inhibitory doses) has been identified as the principal cause of the marked growth in the prevalence of resistance to antibiotics that has taken place over recent decades [18]. Resistance was also observed to nitrofurantoin, a drug that has been banned in the European Union in the 1990s because of its toxicological risks for consumers [82]. Despite the fact that this antimicrobial has not been used on European poultry farms for years, cross-resistance or co-resistance mechanisms could be the cause of the resistance observed to this drug [82]. On the other hand, a very low prevalence of resistance was observed for tetracycline, although it is a compound widely used in European avian farms [81]. The low prevalence of resistance to tetracycline in chicken meat has been observed in previous reports [16]. 

The high prevalence of resistance observed among the strains of the PCR-serogroup IVb is a finding that is coincidental with observations from other researchers [83].

## 4. Material and Methods

### 4.1. Sample Collection

Twenty samples of minced chicken meat, each weighing approximately 400 g, were analyzed. They were procured from various different retail outlets in the cities of León in Spain (ten samples) and of Vila Real in Portugal (ten samples). The chicken meat was minced in the retail outlets. All of the samples were transported in their individual wrappings to the laboratories in the two locations, where they were processed immediately upon arrival.

### 4.2. Counts of Microorganisms Indicating the Quality of Hygiene 

From each sample, 25 g was taken and placed with 225 mL of 0.1% peptone water into a sterile bag with a filter. Homogenization was performed over 120 s in a paddle homogenizer (Stomacher, IUL Instruments, Barcelona, Spain). Decimal dilutions were carried out with the same diluent. Table 4 shows the culture media, the incubation conditions, and the references used for each of the microbial groups studied. All the culture media used in this research were products of the company Oxoid Ltd. (Hampshire, United Kingdom).

### 4.3. Isolation and Identification of Listeria monocytogenes

In detecting *L. monocytogenes*, the method specified by the UNE-EN ISO 11290-1 standard was used. Samples weighing 25 g each were placed in sterile bags with filters and homogenized with an IUL Stomacher in 225 mL of Half Fraser broth for 120 s. After 24 h of incubation at 30 °C, quantities of 10 µL were transferred to tubes with 10 mL of Fraser broth, these being incubated at 37 °C for 24 h. Following this, they were streak-plated on the Oxoid Chromogenic *Listeria* Agar OCLA. A further 48 h of incubation at 37 °C followed, after which five greenish blue colonies with haloes, assumed to be the strains of *L. monocytogenes*, were taken for later identification. The strains were kept at −50 °C in tryptone soya broth (TSB) with 20% glycerol (PanReac AppliChem, Darmstadt, Germany). The culture media were from Oxoid Ltd. (Hampshire, United Kingdom).

The identification of the isolates was carried out by means of a PCR, utilizing primers and conditions specifically for detecting the gene *lmo1030* (Table 5).

The DNA of the strains was extracted from a TSB culture broth incubated for between 18 and 24 h at 37 °C, centrifuged at 13,000 rpm for 60 s twice, and followed by exposure to 100 °C for 30 min in a water bath. The purity and concentration of the DNA were determined using a Nano-Drop One spectrophotometer (Thermo Scientific, Wilmington, Delaware, USA); the wavelength being set at 260 nm. Concentrations of between 80 ng/µL and 180 ng/µL were seen as suitable.

The PCR reactions were carried out in an overall volume of 25 µL, incorporating 5 µL of DNA, 0.5 μM of each primer (Isogen Life Sciences, Barcelona, Spain), a mixture of 0.2 mM of deoxynucleoside triphosphates (dNTPs) obtained from EURx (Gdansk, Poland), a reaction buffer at a 1× concentration (BIORON, Diagnostics GbmH, Ludwigshafen, Germany), MgCl_2_ with a concentration of 3 mM (BIORON), Taq DNA polymerase (BIORON), and sterile distilled water to make up a final volume of 25 µL.

DNA amplification was performed in a thermocycler by Bio-Rad (Hercules, CA, USA). This was programmed as follows: an initial denaturation cycle at 95 °C for five minutes, then 35 amplification cycles (denaturation for 30 s, annealing for 30 s, and elongation at 72 °C for 45 s), followed by a final elongation period of five minutes. Amplification products were separated by means of horizontal electrophoresis on agarose gel (BIORON) at 1% (weight/volume) in a 1× tris-acetate-EDTA buffer stained with SimplySafe (EURx) and diluted to 1:10,000. A loading buffer of glycerol (PanReac AppliChem, Darmstadt, Germany) was used together with a bromophenol blue dye (Panreac Química S.L.U., Barcelona, Spain). In the visualization of the electrophoresis bands an ultra-violet transilluminator (Gel Doc EZ System, Bio-Rad) was used. The size of each PCR product was estimated using markers with a standard molecular weight (10 kb DNA Ladder from BIORON). All the PCR trials included controls, which were both negative (MilliQ water) and positive (*L. monocytogenes* ATCC 13932).

### 4.4. Detecion and Quantification of Listeria monocytogenes by q-PCR

To detect and quantify *L. monocytogenes* in minced chicken by means of a q-PCR, 1.6 mL of the contents of the homogenization bags was taken together with 0.1% peptone water (Oxoid) and deposited in a 5 mL Falcon™ round-bottom tube (Thermo Fisher Scientific, Waltham, MA, USA) and shaken with a vortex agitator. Thereafter, a measured quantity of 750 μL of the contents of the tube was removed. DNA extraction was performed with the aid of a PrepSEQ^TM^ Rapid Spin Sample Preparation Kit with Proteinase K (Thermo Fisher Scientific). For amplification by means of the q-PCR, 30 µL of the extracts of DNA were deposited in each of the wells of a MicroSEQ™ *L. monocytogenes* detection kit (Thermo Fisher Scientific). Both the extraction and amplification of the DNA were performed in accordance with the manufacturer’s instructions.

Subsequently, a quantity of 800 µL was transferred from the Falcon™ tube (Thermo Fisher Scientific) to a sterile Eppendorf tube and a volume of 1 µL of the viability marker PMAxx (Biotium, Fremont, CA, USA) was added, yielding a final dye concentration of 25 µM, this mix being stirred with the pipette. The samples with PMAxx were incubated at 42 °C for 30 min in darkness and manually rotated several times to encourage binding of the colourant to the DNA of the damaged cells. The dyed samples were next exposed to a halogen light source for 15 min at a distance of approximately 20 cm while placed on a block of ice covered in aluminium foil to enhance light reflection. A measured quantity of 750 μL was then taken from the contents of the Eppendorf tube and DNA extraction was carried out using a PrepSEQ^TM^ Rapid Spin Sample Preparation Kit with Proteinase K (Thermo Fisher Scientific). To amplify them using the q-PCR, 30 µL of the extracts of DNA were put into each of the wells of a MicroSEQ™ *L. monocytogenes* detection kit (Thermo Fisher Scientific). Both the extraction and the amplification of DNA were carried out in accordance with the manufacturer’s instructions.

The samples were placed into the thermal cycler of a StepOne^TM^ Real-Time PCR System (Applied Biosystems, Foster City, California, United States); the fluorescence threshold being set at 0.3. To convert amplification cycles into quantities of DNA, a standard straight line (y = −3.0525x + 23.206; R^2^ = 0.966), derived from samples with known amounts of *L. monocytogenes* DNA, was used [87]. To extrapolate the quantity of DNA in terms of bacterial concentrations (log_10_ cfu per gram of sample) the size of the genome of *L. monocytogenes* was considered, where 1 ng of DNA was deemed approximately equivalent to 340,000 cfu [88]. Calculations were performed on the basis of the following equation [87]:

$$L.\;monocytogenes\;\mathrm{concentration}\;\left({\mathrm{Log}}_{10}\frac{\mathrm{cfu}}{g}\right)={\mathrm{Log}}_{10}\;\left(\frac{10^{\frac{\mathrm{Ct}-23.206}{-3.0525}}\times 340,000\times 10^{5}}{750}\right)\mathrm{cfu}/g$$

In establishing this equation, various items were considered. These were: (1) the total volume of the homogenization bag (250 mL, or 250,000 µL), (2) the decimal dilution performed to produce the homogenate (25 g of sample in 225 mL of diluent), (3) the fact that the reaction tube receives one-tenth of the total amount of DNA extracted (30 µL out of 300 µL), and (4) that the DNA was extracted solely from 750 µL.

**Figure 5:**
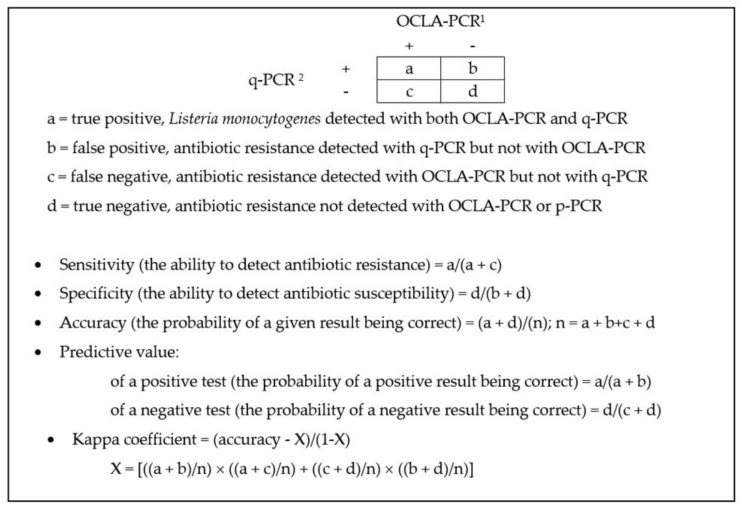
The definition and calculation of the sensitivity, specificity, efficiency, predictive value, and kappa coefficient of the q-PCR for detection of *Listeria monocytogenes* in minced poultry. OCLA–PCR ^1^, isolation by the method specified by the UNE-EN ISO 11290-1 standard (on selective OCLA medium) with identification of the presumptive strains by polymerase chain reaction (PCR); ^2^ detection by quantitative PCR.

In comparing *L. monocytogenes* detection data obtained with the classic method (OCLA–PCR) and those from the q-PCR, the sensitivity, specificity, efficiency, and predictive value of the second method were calculated. Since the degree of contamination of samples was unknown, the calculation method assumed that the conventional OCLA–PCR technique yielded the correct results, acting as a benchmark method. In addition, the two methods were compared by calculating the kappa co-efficient, or agreement [30]. The definitions and way of working out these parameters are shown in Figure 5.

### 4.5. Multiplex PCR Serogrouping of Listeria monocytogenes Isolates

*L. monocytogenes* isolates were further confirmed and classified in PCR groups of serotypes with a multiplex PCR assay, in accordance with the method described by Doumith et al. [89], with minimal modifications. As PCR templates, three to five bacterial colonies grown on tryptone soya agar (TSA) plates (Oxoid) were emulsified in tubes with 50 µL of a solution formed by Tris-HCl 10 mM and EDTA 1 mM (pH = 8.0) and incubated at 99 °C for 15 min. Subsequently, the solutions were cooled in ice and 200 µL of distilled water was added to each mixture. The tubes were centrifuged at 13.000 rpm for 5 min and 50 µL of the supernatant were taken. PCR reactions were carried out incorporating 1 µL of DNA; 1 μM of each of the primers for *lmo0737*, *ORF2819,* and *ORF2110*; 3 μM of the primer for *lmo1118*; 0.2 μM of primer for *prs* (Macrogren Humanizing Genomics, Seoul, Republic of Korea) (Table 6); DNA AmpliTools Multiplex Master Mix 2× (BIOTOOLS, Madrid, Spain); and sterile distilled water to make up a final volume of 20 µL.

The PCR was performed with an initial denaturation step at 94 °C for 3 min, which included 35 cycles of 94 °C for 40 s, 53 °C for 1 min 15 s, and 72 °C for 1 min 15 s; one final cycle of 72 °C for 7 min in a thermocycler ProFlex™ (Applied Biosystems, Waltham, Massachusetts, EEUU). The amplification products (10 µL of the reaction mixture was used) were separated as indicated in previous paragraphs for *L. monocytogenes* identification, but with slight modifications (agarose gel at 2%). Table 7 shows the correlation of multiplex PCR fragment amplification and conventional serotyping.

### 4.6. Antibiotic Susceptibility Tests

The susceptibility of seventy isolates of *L. monocytogenes* (five colonies isolated from the OCLA medium for each positive sample) to a panel of fifteen antibiotics was determined. The disc diffusion method described by the Clinical and Laboratory Standards Institute [90] was employed. Initially, the strains were cultured in tubes holding 9 mL of Mueller Hinton broth (MHB, Oxoid), 20 µL being taken with an inoculation loop from each of the cultures kept frozen. These tubes were incubated for 11 h at 37 °C and their contents were used to inoculate Petri dishes with Mueller Hinton agar (MHA, Oxoid) using a spread plate technique, producing a lawn of culture with a sterile swab. The antibiotic discs were then placed, five on each dish, with sterile tweezers. Antibiotics of 10 categories were tested. These were (1) beta-lactams (ampicillin, AMP, 10 µg; oxacillin, OX, 1 µg; cefoxitin, FOX, 30 µg; cefotaxime, CTX, 30 µg; cefepime, FEP, 30 µg), (2) aminoglycosides (gentamycin, CN, 10 µg), (3) macrolides (erythromycin; E, 15 µg), (4) glycopeptides (vancomycin, VA, 30 µg), (5) sulphonamides (trimethoprim-sulfamethoxazole, SXT, 25 µg), (6) ansamycins (rifampicin, RD, 5 µg), (7) tetracyclines (tetracycline, TE, 30 µg), (8) amphenicols (chloramphenicol, C, 30 µg), (9) fluoroquinolones (ciprofloxacin, CIP, 5 µg; enrofloxacin, ENR, 5 µg), and (10) nitrofuran derivatives (nitrofurantoin, F, 300 µg). All antibiotic discs were purchased from Oxoid.

The plates were incubated for 24 h at 37 °C in an inverted position. The inhibition zones were measured and the strains were classified as sensitive, having reduced sensitivity, or resistant, as a function of the size of the inhibition halo in accordance with the guidelines published by the European Committee on Antibiotic Susceptibility Testing (EUCAST) in 2020 [91] for CN, E, SXT, RD, TE, C, and CIP, and by the CLSI in 2018 [90] for AMP, FOX, CTX, FEP, VA, ENR, and F. 

### 4.7. Statistical Analysis

For the purposes of statistical analyses, microbial counts were converted into logarithmic units (log_10_ cfu/g) and compared using analysis of variance (ANOVA), the means being separated with Duncan’s multiple range test. The prevalence levels of *L. monocytogenes* and of the resistance to antibiotics in the samples from the two countries were compared using Fisher’s exact test. In the comparison of the number of resistances per strain in the various groups of samples, the nonparametric Mann–Whitney U test was used. Significant differences were established with a probability level of 95% (*p* < 0.05). All statistical analyses were performed with the aid of the Statistica^®^ 8.0 software package (StatSoft Inc., Tulsa, OK, USA).

## 5. Conclusions

The results of this research work indicate that chicken mince is a foodstuff of dubious quality from the point of view of hygiene, given the considerable amounts of viable aerobic microbiota, psychrotrophic microorganisms, and enterobacteria found. Moreover, this sort of product provides a large reservoir of strains of *L. monocytogenes* belonging to the PCR-serogroups frequently associated with human listeriosis and showing resistance to multiple antibiotics of clinical importance, a worrying fact in the context of food safety. This entails a need for those handling such foodstuffs to be thoroughly trained in food hygiene, with the aim of avoiding bad practices, like inadequate cooking or cross-contamination, thus reducing the risk to consumers.

The results obtained highlight the limitations of both the classic method for isolating by two-fold enrichment and inoculation onto a selective, differential solid medium, which confirms the bacterial species with a conventional PCR, and also of the q-PCR for detecting *L. monocytogenes* in samples of poultry. The classic method does not permit the detection of *L. monocytogenes* cells that are viable, but not culturable. On the other hand, the q-PCR method is not suitable for detecting low levels of *L. monocytogenes*, in the light of this technique’s high threshold for detection (2.15 log_10_ cfu/g). These findings indicate the usefulness of combining the two techniques (OCLA–PCR and q-PCR) with a view to enhancing the detection of *L. monocytogenes* in food. 

## Figures and Tables

**Table 1:** Microbial counts (log_10_ cfu/g) recorded in samples of minced chicken from Spain and Portugal.

Microbial Group	Origin of the Minced Chicken Samples		
	Spain (*n* = 10)	Portugal (*n* = 10)	All the Samples (*n* = 20)
Viable aerobic microbiota	7.81 ± 0.85 ^a^_a_	7.29 ± 1.12 ^a^_a_	7.53 ± 1.02 _a_
Psychrotrophic microorganisms	7.56 ± 0.86 ^a^_a_	6.64 ± 1.10 ^b^_a_	7.13 ± 1.07 _a_
Enterobacteria	4.55 ± 0.96 ^a^_b_	4.02 ± 0.78 ^a^_b_	4.23 ± 0.88 _b_

Values (average ± standard deviation) in the same row that share any superscript letter have no significant differences one from another (*p* > 0.05). Figures in the same column sharing any subscript letter have no significant differences one from another (*p* > 0.05).

**Table 2:** The transformed results of the quantification of *Listeria monocytogenes* in samples of minced chicken by q-PCR.

SAMPLE		Results with q-PCR ^1^							Detection with OCLA–PCR ^2^
		Total Cells			Viable Cells				
		Ct ^3^	DNA (ng) in the Reaction Tube	Log_10_ cfu/g in the Sample	Ct PMA	DNA (ng) in the Reaction Tube	Log_10_ cfu/g in the Sample	% of Total Cells	
SAMPLES FROM SPAIN	CP1	38.28	0.000012	2.72	40.00	0.000003	2.15	27.3	+
	CP2	>40	<0.000003	<2.15	>40	<0.000003	<2.15	-	+
	CP3	35.26	0.000112	3.71	39.15	0.000006	2.43	5.3	+
	CP4	39.72	0.000004	2.25	39.92	0.000003	2.18	86.0	−
	CP5	>40	<0.000003	<2.15	>40	<0.000003	<2.15	-	+
	CP6	33.40	0.000458	4.32	38.37	0.000011	2.69	2.4	+
	CP7	>40	<0.000003	<2.15	>40	<0.000003	<2.15	-	+
	CP8	>40	<0.000003	<2.15	>40	<0.000003	<2.15	-	+
	CP9	>40	<0.000003	<2.15	>40	<0.000003	<2.15	-	+
	CP10	36.29	0.000052	3.37	37.59	0.000019	2.94	37.5	+
SAMPLES FROM PORTUGAL	CPT1	>40	<0.000003	<2.15	>40	<0.000003	<2.15	-	−
	CPT2	>40	<0.000003	<2.15	>40	<0.000003	<2.15	-	+
	CPT3	>40	<0.000003	<2.15	>40	<0.000003	<2.15	-	−
	CPT4	>40	<0.000003	<2.15	>40	<0.000003	<2.15	-	+
	CPT5	39.53	0.000004	2.31	40.00	0.000003	2.15	70.2	+
	CPT6	>40	<0.000003	<2.15	>40	<0.000003	<2.15	-	−
	CPT7	37.34	0.000023	3.03	40.00	0.000003	2.15	13.5	+
	CPT8	33.93	0.000307	4.14	36.67	0.000039	3.25	12.7	−
	CPT9	37.33	0.000024	3.03	40.00	0.000003	2.15	13.3	−
	CPT10	39.66	0.000004	2.27	40.00	0.000003	2.15	77.4	+

^1^, detection by quantitative PCR. ^2^, OCLA–PCR isolation by the method specified by the UNE-EN ISO 11290-1 standard (on selective OCLA medium) with identification of the presumptive strains by polymerase chain reaction (PCR; *lmo1030* gene); ^3^, Ct = threshold cycle.

**Table 3:** Antibiotic resistance patterns shown by 70 isolates of *Listeria monocytogenes* from Spanish and Portuguese minced chicken samples.

Antibiotic Resistance Pattern	Number of Isolates		
	From Spain	From Portugal	Total
OX-FOX-FEP	0	1	1
OX-FOX-CTX-FEP	1	7	8
OX-FOX-CTX-FEP-SXT	0	1	1
OX-FOX-CTX-FEP-CIP	7	6	13
OX-FOX-CTX-FEP-F	3	3	6
OX-FOX-CTX-FEP-E	1	0	1
OX-FOX-CTX-FEP-RD	1	1	2
OX-FOX-CTX-FEP-SXT-CIP	2	0	2
OX-FOX-CTX-FEP-RD-CIP	3	0	3
OX-FOX-CTX-FEP-CIP-F	10	4	14
OX-FOX-CTX-FEP-E-RD	1	0	1
OX-FOX-CTX-FEP-SXT-CIP-F	2	0	2
OX-FOX-CTX-FEP-RD-CIP-F	5	1	6
OX-FOX-CTX-FEP-E-CIP-F	1	0	1
OX-FOX-CTX-FEP-E-RD-CIP	1	0	1
OX-FOX-CTX-FEP-SXT-TE-CIP-F	0	1	1
OX-FOX-CTX-FEP-CN-E-SXT-CIP	1	0	1
OX-FOX-CTX-FEP-E-RD-CIP-F	1	0	1
OX-FOX-CTX-FEP-SXT-CIP-ENR-F	1	0	1
OX-FOX-CTX-FEP-SXT-RD-CIP-F	3	0	3
OX-FOX-CTX-FEP-E-SXT-RD-TE	1	0	1

OX (oxacillin, 1 µg), FOX (cefoxitin, 30 µg), CTX (cefotaxime, 30 µg), FEP (cefepime, 30 µg), CN (gentamycin, 10 µg), E (erythromycin, 15 µg), SXT (trimethoprim-sulfamethoxazole, 25 µg), RD (rifampicin, 5 µg), TE (tetracycline, 30 µg), CIP (ciprofloxacin, 5 µg), ENR (enrofloxacin, 5 µg), and F (nitrofurantoin, 300 µg).

**Table 4:** Culture media, incubation conditions, and references for each microbial group studied.

Microbial Group	Culture Media	Incubation		Reference
		Time	Temperature (°C)	
Viable aerobic microbiota	PCA ^1^	72 h	30 °C	[35]
Psychrotrophic microorganisms	PCA ^1^	10 days	7 °C	[84]
Enterobacteria	VRBGA ^2,3^	24 h	37 °C	[85]

^1^ plate count agar; spread-plate technique (0.1 mL); ^2^ crystal violet, neutral red, bile salts, glucose, and agar; pour-plate technique (1 mL); ^3^ with overlay. Inoculations were performed in duplicate.

**Table 5:** The gene and primers used to identify strains of *Listeria monocytogenes* by PCR [86].

Gene	Primer	Sequence (5′ → 3′)	Temperature (°C)	Size (bp)
*lmo1030*	Lmo1030-F	GCTTGTATTCACTTGGATTTGTCTGG	62	509
	Lmo1030-R	ACCATCCGCATATCTCAGCCAACT		

**Table 6:** Nucleotide sequences of the primer sets used in this study [89].

Gene Target	Primer Sequence (5′ → 3′)	Product Size (bp)	Serovar Specificity
*lmo0737*	F: AGGGCTTCAAGGACTTACCC R: ACGATTTCTGCTTGCCATTC	691	*L. monocytogenes* serovars 1/2a, 1/2c, 3a, and 3c
*lmo1118*	F: AGGGGTCTTAAATCCTGGAA R: CGGCTTGTTCGGCATACTTA	906	*L. monocytogenes* serovars 1/2c and 3c
ORF2819	F: AGCAAAATGCCAAAACTCGT R: CATCACTAAAGCCTCCCATTG	471	*L. monocytogenes* serovars 1/2b, 3b, 4b, 4d, and 4e
ORF2110	F: AGTGGACAATTGATTGGTGAA R: CATCCATCCCTTACTTTGGAC	597	*L. monocytogenes* serovars 4b, 4d, and 4e
*prs*	F: GCTGAAGAGATTGCGAAAGAAG R: CAAAGAAACCTTGGATTTGCGG	370	All *Listeria* species

**Table 7:** The correlation of multiplex PCR and conventional serotyping for *Listeria monocytogenes* strains.

Multiplex PCR Fragment Amplification					Serogroup	*Listeria monocytogenes* Serovar	Control Strain
*lmo1118* (906 bp)	*lmo0737* (691 bp)	*ORF2110* (597 bp)	*ORF2819* (471 bp)	*prs* (370 bp)			
−	+	−	−	+	IIa	1/2a, 3a	ATCC ^1^ 19111 (serovar 1/2a)
−	−	−	+	+	IIb	1/2b, 3b, 7	STCC ^2^ 936 (serovar 1/2b)
+	+	−	−	+	IIc	1/2c, 3c	STCC 938 (serovar 3c)
−	−	−	−	+	IVa	4a, 4c	ATCC 19114 (serovar 4a)
−	−	+	+	+	IVb	4b, 4d, 4e	ATCC 13932 (serovar 4b)

^1^ American Type Culture Collection; ^2^ Spanish Type Culture Collection.

## Data Availability

Not applicable.
